# NLP-enhanced inflation measurement using BERT and web scraping

**DOI:** 10.3389/frai.2025.1520659

**Published:** 2025-04-09

**Authors:** Martin Berki, Vanesa Andicsova, Milos Oravec

**Affiliations:** Faculty of Electrical Engineering and Information Technology, Slovak University of Technology in Bratislava, Bratislava, Slovakia

**Keywords:** inflation measurement, natural language processing, web scraping, BERT, price index, economic analysis, HICP

## Abstract

In this research note, we explore the integration of natural language processing (NLP) and web scraping techniques to develop a custom price index for measuring inflation. Using the Harmonized Index of Consumer Prices (HICP) as a benchmark, we created a database of consumer electronics product data through web scraping. Using the BERT model for classification, we achieved a high-performance classification of approximately 10,000 items into COICOP categories, with an accuracy of 94.56 %, macro precision of 79.41 %, and weighted precision of 94.07 % on validation data. Our custom index, particularly with weighted and median methodologies, demonstrated closer alignment with the official HICP while capturing more detailed price fluctuations within the market. Monthly inflation trends revealed variability that reflects price changes in the COICOP 091 category, contrasting with the relative stability of the official HICP. This work provides an alternative perspective on inflation measurement, highlighting the potential of computational approaches to enhance economic analysis.

## 1 Introduction

Inflation measurement is critical for understanding economic trends and guiding policy decisions, investments, and consumer behavior (Diewert et al., 2009; Bryan and Cecchetti, 1993). Traditionally, inflation is measured through the Consumer Price Index (CPI), which monitors price changes in a basket of goods and services (White, 1999). However, manual data collection methods for inflation analysis are limited in frequency and speed, hindering real-time insights (Modugno, 2013).

Advances in technology, particularly web scraping and NLP, offer innovative ways to improve inflation measurement. Web scraping allows for the automated collection of price data from online sources, capturing up-to-date market trends. When combined with NLP, these methods can classify vast amounts of unstructured data–such as product descriptions–into economic categories, such as the Classification of Individual Consumption According to Purpose (COICOP).

In this context, our central research question emerges: can a web-scraped, NLP-based system measure inflation at higher frequencies to capture short-term price fluctuations that are often missed by traditional, less frequent data collection methods? By exploring this question, we aim to determine whether real-time or near-real-time inflation tracking is feasible and how it compares to official indices.

This study presents a system for analyzing inflation within the consumer electronics market. Our approach uses web scraping methods to collect market data, a structured database to organize it, and comparative analysis against official statistics like Eurostat's HICP. We systematically collected data from online retailers and categorized products using the Bidirectional Encoder Representations from Transformers (BERT) model, with the goal of product COICOP classification. Our analysis reveals that indices derived from this data exhibit greater dynamism and price volatility than the HICP, particularly when weighted or median indices are used.

## 2 Related work

Recent advancements in inflation analysis have leveraged innovative methodologies, including web scraping and NLP, to enhance the precision and timeliness of consumer price tracking. These methods have been applied effectively within the framework of the COICOP classification, enabling more granular and actionable insights.

Macias and Stelmasiak (2019) demonstrated the potential of web scraping to improve food inflation nowcasting by integrating online price indices into forecasting models. Their work highlighted the importance of using distributed-lag models to aggregate predictions at the lowest level of disaggregation, aligning closely with COICOP categories.

Benedetti et al. (2022) explored the use of web scraping to compute high-frequency sub-national CPI estimates, demonstrating its utility in identifying regional price trends and seasonal patterns. Their work underscored the value of web-scraped data for supplementing traditional price statistics, particularly when applied to COICOP-classified products.

Aparicio and Bertolotto (2017) provided further evidence of the superiority of web scraping over traditional data collection methods. Their study showed that online price data significantly enhance CPI forecasting models, offering policymakers timely insights and addressing delays inherent in official CPI releases.

Together, these studies showcase the transformative impact of combining web scraping and NLP techniques on inflation analysis. By aligning these methods with COICOP categories, researchers have enabled more granular, accurate, and actionable insights, advancing both academic and policy applications in the field.

## 3 Method

### 3.1 Web scraping process

Web scraping automates data extraction from web pages by sending HTTP requests, receiving HTML responses, and parsing useful information into databases for analysis (Diouf et al., 2019). This technique efficiently gathers large volumes of web data essential for real-time, structured analysis.

We developed a web scraper to systematically extract product information from a popular Slovak price comparison portal that provides access to historical product prices. By parsing each item's current price, price history, product name, and parameters, our scraper offers detailed insights into price trends over time in the consumer electronics market.

The scraper was deployed on an Ubuntu server and scheduled to run daily via a cron job. Given that many modern websites generate content dynamically via JavaScript, we employed Selenium to accurately render pages and handle any embedded scripts. We then used BeautifulSoup for efficient and intuitive HTML parsing once the content was fully loaded. This combination allowed us to balance flexibility and ease of implementation, ensuring that both dynamic and static webpage components were consistently retrieved. The extracted data, including product parameters like brand, model, and features, was stored in a PostgreSQL database. This structured database serves as the foundation for the further classification and analysis, reflecting current market conditions and historical price trends.

Regarding ethical considerations, we began by reviewing the portal's robots.txt file to confirm which sections of the site were permissible to crawl. We also implemented a rate limiter to space out our requests, thus minimizing load on the server and ensuring compliance with the site's usage policies.

### 3.2 Dataset

We gathered nearly 10,000 samples from the consumer electronics sector, where samples consisted of product descriptions, parameters, and price histories. These data were obtained from a price comparison portal tracking prices of products across multiple stores. Sample from dataset can be seen in Listing 1.

Listing 1 **Sample from dataset represented by JSON file. Data has predefined structure with name and category attributes, alongside with mean, and minimum price recordings across different dates. The length of price records is based on data availability from that date, with possibility of retrieval historical prices. Data also have nested JSON with product parameters, where structure (set of parameters) is dynamic, based on product description on price-portal. Based on these parameters, are products later classified into COICOP hierarchy.**


    {
        **"name"**: "Philips 32HFL3014",
        **"category_name"**: "LED TV (LCD) - E-shop",
        **"COICOP"**: 9101203,
        **"parameters"**: {
            "DVB-C": "yes",
            **"DVB-T2"**: "yes",
            **"Weight"**: "4.6 kg",
            **"DVB-T tuner"**: "yes",
            **"Resolution"**: "1366 x 768",
            **"TV Type"**: "LED",
            **"Number of HDMI inputs"**: "1",
            **"Headphone output"**: "yes",
            **"Screen size"**: "81.3 cm",
            **"Screen size (inches)"**: "32 inches"
        },
        **"dates"**: "2019-10-28,2019-11-04,2019-11-18,2019-11-25**",**
        **"**price_min**": "**438.0,417.84,371.28,371.28,471.42,510.52,471.42**",**
        **"**price_avg**": "**438.0,427.47,451.53,451.07,490.64,510.52,489.53**"**
    **}**

The dataset was annotated according to the guidelines from the Slovak Statistical Office, which categorizes products into the COICOP hierarchy. Approximately half of the samples did not meet the criteria for inclusion in the official price index and were classified into a general category. This resulted in a dataset imbalance that was addressed during the classification phase. The final dataset was stored in JSON format, with products encoded by integers representing their COICOP categories.

Annotation was performed semi-automatically, where we filtered products in source webpage based on their attributes defined by Statistical Office. For example, if Statistical Office takes into account only LCD monitor with specific resolution and screen size, we filtered only products that match this criteria. These products were then placed into their COICOP category. Then we turned off product filters and we listed all products from current explored category (in this example LCD monitors). We deleted all product that were listed in previous step (suitable products) and all remaining products were labeled as unsuitable or unclassifiable for COICOP. These were also used for training our model. Based on variety of products, model better understands their differences and nuances of product parameters that are key for COICOP classification compliance.

As seen in Table 1, the majority of products in our dataset were classified as “Unclassifiable into COICOP” (5,481 records). This was because many items, such as headphones, tablets, and TVs, did not meet the strict criteria set by the statistical office for COICOP classification. As a result, these products were grouped into the unclassifiable category, reflecting the challenges of aligning real-world product diversity with standardized classification frameworks.

**Table 1:** Product counts for each COICOP category in our dataset.

**Category name**	**COICOP**	**Counts**
Unclassifiable into COICOP	N/A	5,481
Headphones—over head, with cable	9101105	979
Notebook	9103102	638
Hard-drive	9104107	463
Personal computer (no accessories)	9103101	424
Memory stick	9104104	324
LED TV (LCD) - E-shop	9101203	310
LCD (LED) monitor 27–32 inches	9103108	262
Ultra HD (4K) LED TV	9101201	164
USB stick	9104106	138
Tablet	9103107	133
Electronic pocket calculator	9103103	90
Mini-system (micro-system)	9101103	68
Empty DVD	9104102	55
Digital compact—E-shop	9102104	41
Car camera	9102102	37
Portable radio with CD/MP3	9101101	34
Empty compact disc (CD-R)	9104103	32
Color multifunction printer	9103105	16
MP4 Player—E-shop	9101104	13
SLR Camera (body plus lens)	9102103	12
Software—operating system	9103106	6

**Figure 1:**
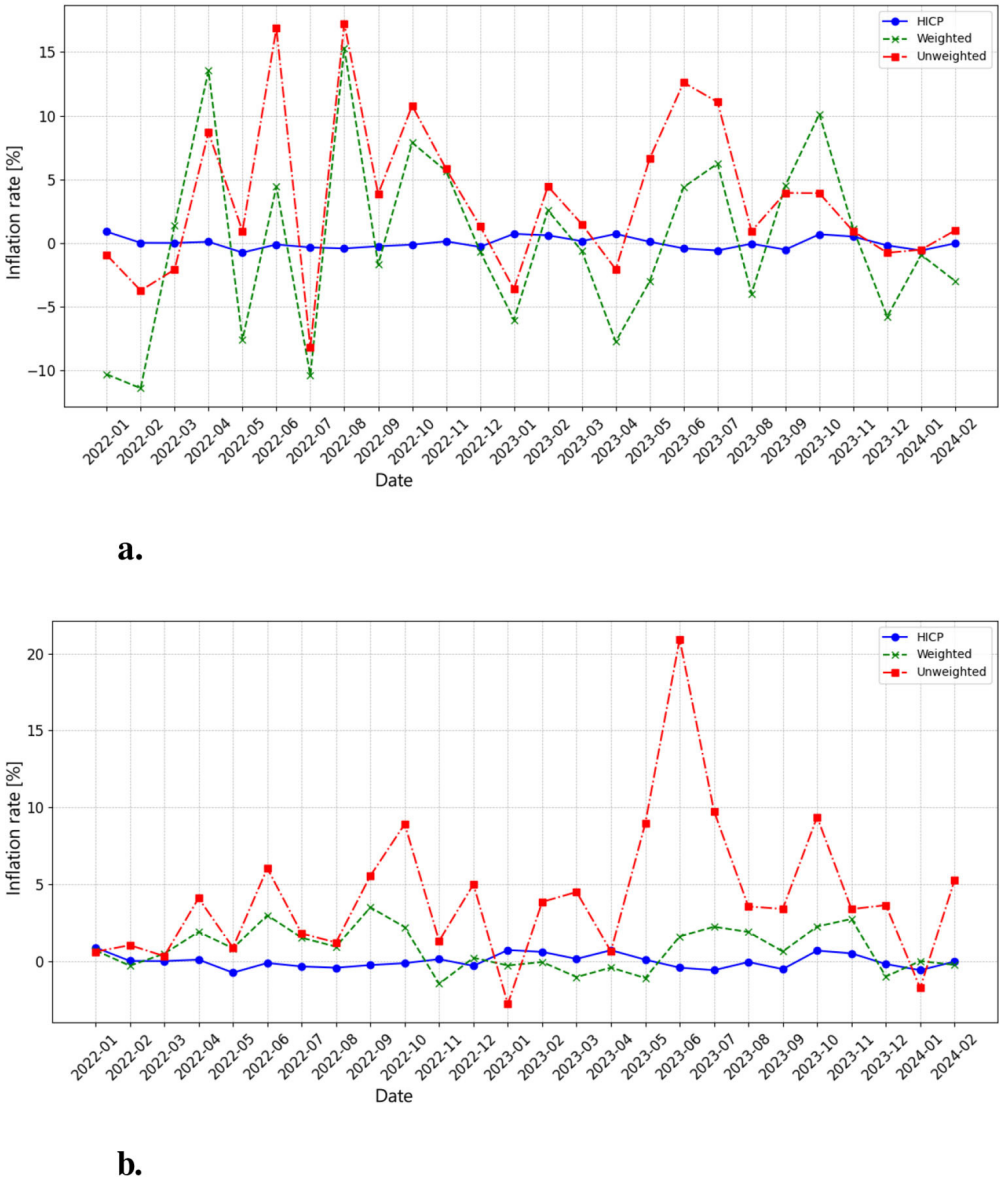
**(a)** This chart presents the monthly inflation rate for COICOP 091 (Audio, Video, and Processing Equipment), calculated from the arithmetic mean of product prices. The HICP inflation rate (blue line) remains relatively stable around zero, indicating that official consumer price inflation for this category has experienced minimal fluctuations. In contrast, the unweighted inflation rate (red line) and weighted inflation rate (green line) show high volatility, with frequent peaks and troughs exceeding +15% and falling below -10%. This volatility suggests that real market prices are subject to frequent short-term fluctuations, likely driven by product releases, discount cycles, and supply chain shifts. **(b)** This chart presents the monthly inflation rate for COICOP 091, calculated from the median of product prices. Compared to the mean-based calculation, the median-derived inflation rates are more stable, with fewer extreme negative fluctuations. However, the unweighted inflation rate (red line) still shows strong spikes, exceeding 20% in mid-2023, indicating broad-based price increases across product categories. The weighted inflation rate (green line) follows a smoother trajectory, reducing volatility while still capturing periods of moderate inflation. The HICP inflation rate (blue line) remains stable, reinforcing the contrast between traditional inflation measures and real-time price dynamics from web-scraped data. The median-based approach reduces sensitivity to extreme price changes, offering a more balanced view of underlying inflation trends.

The dataset also presented several nuances that impacted the distribution of records across categories. For instance, the statistical office's criteria only cover SLR cameras, while mirrorless cameras, which are now more popular, were not included (Yoon and Anderson, 2014; Chitra et al., 2024). This led to fewer samples being collected for the SLR camera category. Additionally, the BERT model often misclassified SLR cameras as digital compact cameras due to the similarity in their parameters, as evidenced in the confusion matrix (Figure 1).

Another notable limitation was observed in the operating systems category. Only Windows 10 met the statistical office's criteria, which significantly restricted the number of samples gathered for this category. This shows how difficult is capturing a representative sample when classification criteria are overly narrow or outdated.

Most samples were collected for categories such as headphones, notebooks, and tablets, which are more commonly available and better aligned with the COICOP framework. In contrast, other categories, such as mini-systems, portable radios, and SLR cameras, had fewer than 100 records (products) in the database. This uneven distribution underscores the challenges of building a comprehensive dataset that reflects both market trends and the constraints of standardized classification systems.

### 3.3 Classification of product descriptions

For the classification task, we fine-tuned the BERT Base uncased model, which consists of approximately 110 million parameters (Devlin et al., 2019). Developed by Google AI in 2018, BERT (Bidirectional Encoder Representations from Transformers) is based on the transformer architecture and demonstrated significant advancements in various natural language processing tasks shortly after its release, from extractive text summarization (Liu, 2019) to language-specific adaptation for Dutch (de Vries et al., 2019) and multilingual applications (Nozza et al., 2020).

We opted for BERT due to its proven effectiveness in text classification tasks and its capacity to handle extensive and specific product descriptions. While simpler methods (e.g., traditional machine learning algorithms or basic embedding techniques) could have been considered, our goal was to explore a modern deep learning approach that captures context bidirectionally. BERT Base uncased provided a balance between model complexity and ease of integration with established frameworks, making it well-suited for our classification needs. This choice allowed us to leverage pre-trained transformer representations and fine-tune them efficiently for the specific nuances of the consumer electronics domain.

We trained the model for 10 epochs with a batch size of 32, using a weight decay of 0.01 and ADAM as the optimizer. The dataset was split into 80 % for training and 20 % for validation.

Product descriptions served as the input text data in JSON format, which were processed using the BERT tokenizer to convert the text into a suitable format for the model. The fine-tuning process involved adjusting the pre-trained BERT model weights specifically for our classification task, enabling the model to learn the nuances of categorizing products into multiple classes from audio-video category.

### 3.4 Price Index estimation in the COICOP hierarchy

Price indices measure changes in the price level of a basket of goods and services over time, and they are essential for understanding inflation. In the context of the COICOP hierarchy, price indices are calculated hierarchically (Eurostat, 2018). This means that the price index of a broader category (e.g., “Food and non-alcoholic beverages”) is derived from the price indices of its subcategories (e.g., “Bread and cereals,” “Meat,” etc.).

At the lowest level of the COICOP hierarchy (level 6), official Harmonized Index of Consumer Prices (HICP) values are not directly provided by Eurostat. Instead, HICP data is available starting from level 5. To address this gap, we use statistical methods to estimate the price index at level 6. Specifically, we calculate either the mean or the median of the available data points to represent the price index at this level. These measures provide a reasonable approximation when official data is unavailable.

The official HICP price index is computed using a weighted average, where each subcategory's price index is multiplied by its corresponding weight, reflecting its relative importance in household consumption. These weights are based on official expenditure data, ensuring that the index accurately reflects market trends and consumer behavior. For example, if “Housing” has a larger weight than “Recreation,” changes in housing prices will have a greater impact on the overall index.

In addition to the weighted index, we also calculate an unweighted index using a simple average of the subcategory price indices. This unweighted index serves as a useful comparison tool, allowing us to assess how the weighting scheme influences the measured evolution of inflation. By comparing the weighted and unweighted indices, we can better understand whether changes in inflation are driven by specific high-impact categories or are more evenly distributed across all categories.

### 3.5 Price index and inflation rate calculation

To calculate the price index, we first computed a simple average of product prices within the COICOP 091 level 6 categories, aggregating these gradually to level 3. We selected December 2021 as the base period because it marks a critical structural break in inflationary dynamics, as identified by Ball et al. (2022) in empirical studies of post-pandemic economies in document from National Bureau of Economic Research—Working Paper 30613. This period coincides with the convergence of several macroeconomic forces: the delayed effects of unprecedented fiscal stimulus (e.g., the U.S. American Rescue Plan), global supply chain reconfigurations, and geopolitical tensions that disrupted energy markets. As shown in this document, late 2021 saw a sharp inflection in labor market tightness (measured by the V/U ratio), which rose from 0.9 in early 2021 to over 2.0 by March 2022, directly amplifying wage and price pressures. By anchoring the index to this period (December 2021 = 100), we isolate subsequent inflationary shocks against a baseline that reflects the “new normal” of post-COVID economic conditions. This methodology also mirrors advanced-economy practices, such as U.S. analyses linking the 2021-2022 inflation surge to intersecting demand-pull and cost-push factors.

The price index for each given period *t* was computed using the equation:


(1) $$\begin{array}{l} {\text{Index}}_{t}=\left(\frac{{\text{Price}}_{t}}{{\text{Price}}_{\text{base}}}\right)\times 100 \end{array}$$

where Price_*t*_ is the average price in period *t*, and Price_base_ is the price in the base period. This ensures that the index reflects price changes relative to the baseline, which is always normalized to 100.

In addition to the simple average method, we also calculated a median-based index as a non-official methodology, providing an alternative perspective on price dynamics. Across levels 6 to 3 of the COICOP hierarchy, we considered a total of 21 categories for price index aggregation.

The inflation rate was derived from the computed price indices. Specifically, the inflation rate for period *t*, denoted as π_*t*_, was calculated as the percentage change in the Harmonized Index of Consumer Prices (HICP) between two consecutive periods:


(2) $$\begin{array}{l} \pi_{t}=\frac{{\text{HICP}}_{t}}{{\text{HICP}}_{t-1}}-1 \end{array}$$

Equation 2 measures the proportional increase or decrease in the price level from one period to the next, thus capturing the inflation dynamics over time.

## 4 Results

### 4.1 Training results

The classification of products into COICOP categories yielded high performance across several key evaluation metrics. The fine-tuned BERT model achieved an overall **accuracy of 94.56%**, with a **cross-entropy loss of 0.17**. Both **macro** and **weighted** versions of **precision, recall, and F1-score** were evaluated to address dataset imbalances. The **weighted precision** reached **94.07%**, while the **macro precision** was lower at **79.41%**, reflecting the challenge of correctly classifying underrepresented categories. Similarly, **recall** was **94.56%** when weighted by class frequency but dropped to **80.84%** in the macro-average. The **F1-score** followed the same trend, achieving **94.03%** in the weighted version and **79.11%** in the macro version.

The **macro metrics** provide an average performance across all classes, treating each class equally regardless of its prevalence. This metric is particularly valuable in evaluating how well the model handles underrepresented classes. In contrast, the **weighted metrics** account for class imbalances by weighting each class's contribution according to its frequency in the dataset. This dual evaluation approach provides a more detailed view of the model's performance across both frequent and infrequent classes, ensuring the robustness of the model's predictions (Mortaz, 2020).

The tuning process for the BERT model lasted for **10 epochs**, with the **best performance observed at epoch 7**, suggesting that additional training beyond this point led to slight overfitting. The slight decline in performance in later epochs indicates reduced generalizability to unseen data.

While the model performs exceptionally well on the **weighted metrics**, reflecting strong accuracy for the more frequent classes, the **macro metrics** reveal some performance variability across less frequent classes. This slight bias toward the more prevalent classes is an expected consequence of the **dataset's imbalance**, but overall, the model's performance remains **robust and reliable** for the classification of products into COICOP categories.

**Figure 2:**
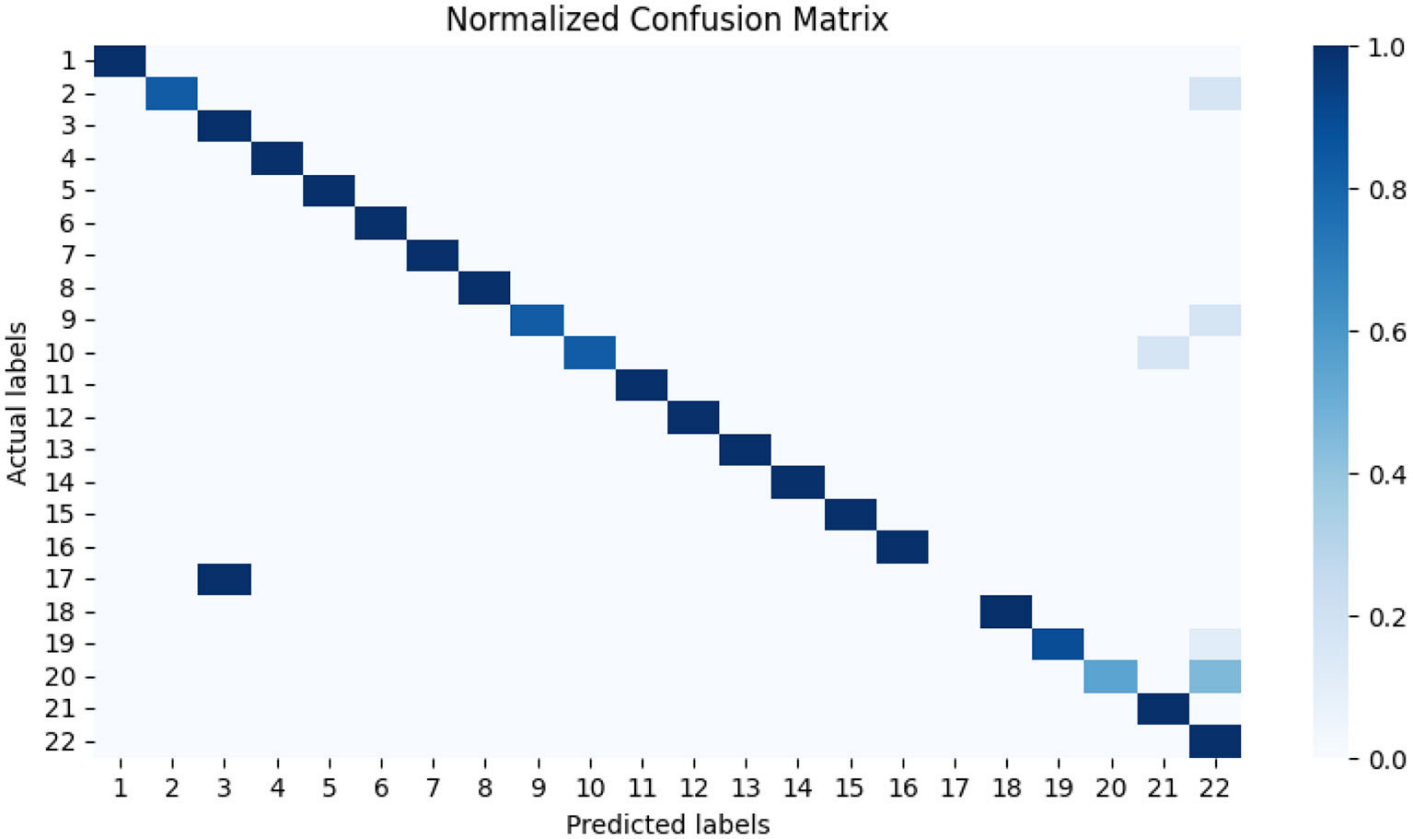
Normalized confusion matrix generated from the validation data provides a deeper insight into the classification outcomes. The normalization helps account for the dataset's imbalances, allowing for a clearer assessment of misclassifications. Label numbers are resolved in following way: 1. Car camera 2. Color multifunction printer 3. Digital compact—E-shop 4. Electronic pocket calculator 5. Empty DVD 6. Empty compact disc (CD-R) 7. Hard-drive 8. Headphones—over head, with cable 9. LCD (LED) Monitor 27–32 inches 10. LED TV (LCD)—E-shop 11. MP4 player—E-shop 12. Memory stick 13. Mini-system (micro-system) 14. Notebook 15. Personal computer (no accessories) 16. Portable radio with CD/MP3 17. SLR Camera (body plus lens) 18. Software—Operating system 19. Tablet 20. USB stick 21. Ultra HD (4K) LED TV 22. Other (unclassifiable).

The normalized confusion matrix in Figure 2 reveals a strong overall classification performance, as evidenced by the majority of diagonal elements being close to 1. This suggests that the model correctly identifies most product categories. However, some misclassifications are notable and can largely be attributed to category similarities and dataset imbalances.

One of the most significant misclassification trends is observed in class 17 (SLR Camera), which is frequently confused with class 3 (Digital Compact Camera). Given the visual and functional similarities between these two product types, combined with the stark imbalance in their representation—only 12 instances of SLR Cameras compared to 41 Digital Compact Cameras–the model struggles to establish clear distinguishing features, leading to frequent misclassifications.

Another major source of error is class 20 (USB Stick), where 45 % of instances are misclassified as class 22 (Unclassifiable into COICOP). This issue likely arises due to the overwhelming presence of unclassifiable products in the dataset (5,481 instances), making it a dominant category that absorbs ambiguous or less-represented items. Similarly, 17 % of class 2 (Color Multifunction Printers) and class 9 (LCD Monitors, 27–32 inches) are also incorrectly classified as unclassifiable, suggesting that these categories lack sufficient sample size or distinguishing characteristics within the dataset to ensure reliable classification.

Furthermore, class 10 (LED TV) exhibits a 17 % misclassification rate with class 21 (Ultra HD 4K LED TV), likely due to the inherent similarity between these two categories. Since Ultra HD TVs are a subset of LED TVs, the distinction between standard LED models and higher-resolution versions may not always be clear in the available training data, causing occasional overlap in classification.

Despite these misclassification trends, the model demonstrates strong classification capabilities across most categories. The observed errors are primarily driven by dataset imbalances and the challenge of distinguishing between closely related product types. Nevertheless, the overall accuracy remains sufficient for practical application in COICOP product classification, with potential for further refinement through additional data collection, feature engineering, or class balancing techniques.

### 4.2 Analysis results

#### 4.2.1 Index analysis results

**Figure 3:**
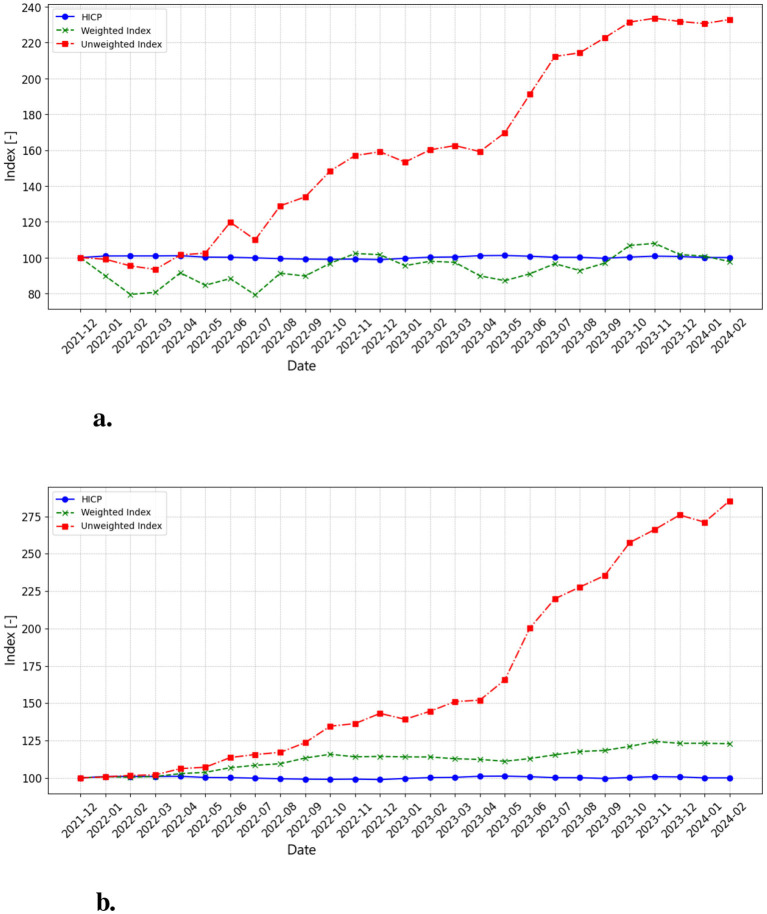
**(a)** This chart displays the monthly price index for COICOP 091 (Audio, Video, and Processing Equipment), computed using the arithmetic mean of web-scraped product prices. The unweighted index (red line) shows a strong and accelerating upward trend, rising from 100 in December 2021 to over 220 by early 2024, indicating substantial price increases in this category. The weighted index (green line) fluctuates between 80 and 100, reflecting short-term volatility but without a sustained increase. Meanwhile, the HICP (blue line) remains stable around 100, suggesting a stark contrast between official inflation measures and raw market prices. **(b)** This chart represents the monthly price index for COICOP 091, calculated using the median instead of the average. The unweighted index (red line) follows a more gradual but ultimately stronger upward trajectory, surpassing 280 by early 2024, indicating a broad-based price increase across all product categories rather than just a few expensive models pulling the index upward. The weighted index (green line) also demonstrates a clear rising trend, reaching approximately 125, suggesting that price growth is not limited to outliers but is affecting the entire market. The HICP (blue line) remains relatively stable at 100, reinforcing the discrepancy between official inflation data and web-scraped indices. The steeper increase in the median-based index suggests that price inflation in consumer electronics is widespread and systematic, rather than being an artifact of extreme price fluctuations in individual high-end products.

The HICP index (blue line) in Figures 3a, b remains stable throughout the observed period, maintaining a base value close to 100. This suggests that, on average, the official prices of audio-video and information processing equipment have remained relatively unchanged. However, both the weighted (green) and unweighted (red) indices exhibit different behavior. Initially stable, the unweighted index begins a clear and sustained upward trajectory from mid-2022, accelerating further between 2023 and early 2024. This trend signals increasing average prices in this COICOP category. The weighted index, designed to account for market share and sales volume, fluctuates less but still captures periods of price growth, mitigating the impact of high-cost but less frequently sold items.

A key distinction between the average-based and median-based indices is the magnitude of inflation captured. While both unweighted indices show significant price increases, the median-based index rises even more steeply, surpassing 280 by early 2024 compared to 220 in the average-based version. This indicates that price inflation is not simply driven by a handful of premium products but rather affects a broader range of items. The weighted index presents different behaviors depending on the calculation method. In the average-based approach, it fluctuates within a narrow range, suggesting that different product categories offset each other. However, in the median-based approach, the weighted index follows a clearer upward trend, reaching approximately 125 by early 2024, implying that price growth is affecting the majority of products.

The increasing gap between the weighted and unweighted indices, particularly in the median-based approach, shows the importance of considering market share and sales volume when constructing a price index. The sharp rise in the median-based unweighted index suggests a broad price surge across categories, reinforcing concerns that traditional inflation indices may understate actual consumer price increases in the technology sector.

#### 4.2.2 Inflation analysis results

The inflation rate calculation based on our own indices provides further insights into the economic dynamics within COICOP 091. By deriving inflation rates from both weighted and unweighted indices, we offer an alternative perspective on price trends compared to those calculated using the HICP.

The comparison between mean-based and median-based inflation rates highlights key methodological differences. The mean-based inflation rate exhibits greater volatility, reflecting sharp price swings and negative corrections. By contrast, the median-based inflation rate smooths out extreme values, offering a more stable measure of inflationary pressure. While both methods indicate periodic price surges and corrections, the HICP remains stable, suggesting that official inflation metrics may not fully capture real-world price fluctuations in this category.

## 5 Discussion

This work demonstrates the potential of integrating NLP and web scraping to enhance inflation measurement within the COICOP 091 category, which includes audio-video and information processing equipment. By collecting a diverse range of products across multiple retailers, our custom inflation index captures price shifts more sensitively than the official HICP, which is limited to a smaller selection of branded items.

Our findings reveal significant differences between inflation rates calculated from our indices and those from the HICP. The HICP inflation shows minimal volatility, suggesting stability in this category over the observed period. In contrast, our custom indices–especially the unweighted index–exhibit greater volatility, with fluctuations ranging from -10 % to +15 % at various points. This difference is likely due to the more frequent data collection in our methodology, as well as the inclusion of a wider array of products, allowing our indices to capture rapid changes in response to factors like seasonal sales, technological advancements, and shifts in consumer demand.

The comparison between weighted and unweighted indices, as well as between average and median-based calculations, underscores the importance of index methodology. Weighted indices smooth out volatility by adjusting for product popularity, while unweighted indices reflect raw price changes, revealing more intense fluctuations. Similarly, median-based indices offer smoother trends by reducing the influence of outliers, while average-based indices can highlight the impact of high-priced items.

### 5.1 Broader implications and practical applications

The ability to construct inflation indices from high-frequency web-scraped data has significant implications for economic policymaking and official statistics. Traditional inflation measurement relies on manually collected price samples, which are updated monthly or less frequently. Our approach, by contrast, enables near real-time inflation tracking. This could help policymakers and central banks detect price fluctuations more quickly, improving inflation forecasting models and allowing for more timely interventions.

Beyond the consumer electronics sector, this methodology could be applied to other areas such as food prices, housing costs, or fuel price monitoring. Web scraping provides the flexibility to capture price dynamics across various industries, making it a valuable tool for enhancing inflation measurement in fast-changing markets. For example, high-frequency online data collection could provide early indicators of rising food prices, improving food security assessments and policy responses. Tracking rental and property prices from listing platforms could supplement official real estate inflation indices, offering more up-to-date insights into housing affordability. Continuous monitoring of fuel prices could help policymakers assess real-time energy inflation, informing strategic interventions in response to oil price fluctuations.

### 5.2 Challenges and future research

Despite its advantages, this approach also presents challenges, including dataset imbalances and the potential for classification errors in underrepresented categories. The large presence of “unclassifiable” products in our dataset (Table 1) highlights the difficulty of aligning real-world product diversity with standardized classification frameworks. Future research could explore techniques such as active learning, improved feature engineering, or collaboration with official statistical agencies to refine classification accuracy.

Another challenge lies in data accessibility and compliance. Web scraping, while effective, may be restricted by website policies, requiring alternative data acquisition strategies. Partnering with e-commerce platforms or leveraging structured datasets from retailers could enhance data reliability while ensuring compliance with ethical considerations.

Furthermore, integrating machine learning with economic analysis opens new possibilities for refining price indices. Future work could explore the use of large language models beyond BERT to improve classification accuracy; expansion to additional product categories and economic sectors and integration of real-time economic event detection to analyze the impact of market shocks on inflation.

## Data Availability

The raw data supporting the conclusions of this article will be made available by the authors, without undue reservation.
